# Reproductive health and quality of life of young Burmese refugees in Thailand

**DOI:** 10.1186/1752-1505-4-5

**Published:** 2010-03-25

**Authors:** Marie T Benner, Joy Townsend, Wiphan Kaloi, Kyi Htwe, Nantarat Naranichakul, Saowalak Hunnangkul, Verena I Carrara, Egbert Sondorp

**Affiliations:** 1 Independent Researcher, 152 Wireless Road, Indosuez House 4th floor, 10330 Bangkok, Thailand; 2 London School of Hygiene and Tropical Medicine, Public and Environmental Health Research Unit, Keppel Street, London,WC1E 7HT, UK; 3 Faculty of Medicine, Siriraj Hospital, Mahidol University, 2 Prannok Road, 10700 Bangkok, Thailand; London School of Hygiene and Tropical Medicine, Non-Communicable Disease Epidemiology Unit, Keppel Street; London, WC1E 7HT, UK; 4 SHOKLO Malaria Research Unit, PO Box 46, Mae Sot, Tak, Thailand; 5 London School of Hygiene and Tropical Medicine, Public Health and Policy Unit London School of Hygiene and Tropical Medicine, Keppel Street; London, WC1E 7HT, UK

## Abstract

**Background:**

Of the 140 000 Burmese* refugees living in camps in Thailand, 30% are youths aged 15-24. Health services in these camps do not specifically target young people and their problems and needs are poorly understood. This study aimed to assess their reproductive health issues and quality of life, and identifies appropriate service needs.

**Methods:**

We used a stratified two-stage random sample questionnaire survey of 397 young people 15-24 years from 5,183 households, and 19 semi-structured qualitative interviews to assess and explore health and quality of life issues.

**Results:**

The young people in the camps had very limited knowledge of reproductive health issues; only about one in five correctly answered at least one question on reproductive health. They were clear that they wanted more reproductive health education and services, to be provided by health workers rather than parents or teachers who were not able to give them the information they needed. Marital status was associated with sexual health knowledge; having relevant knowledge of reproductive health was up to six times higher in married compared to unmarried youth, after adjusting for socio-economic and demographic factors. Although condom use was considered important, in practice a large proportion of respondents felt too embarrassed to use them. There was a contradiction between moral views and actual behaviour; more than half believed they should remain virgins until marriage, while over half of the youth experienced sex before marriage. Two thirds of women were married before the age of 18, but two third felt they did not marry at the right age. Forced sex was considered acceptable by one in three youth. The youth considered their quality of life to be poor and limited due to confinement in the camps, the limited work opportunities, the aid dependency, the unclear future and the boredom and unhappiness they face.

**Conclusions:**

The long conflict in Myanmar and the resultant long stay in refugee camps over decades affect the wellbeing of these young people. Lack of sexual health education and relevant services, and their concerns for their future are particular problems, which need to be addressed. Issues of education, vocational training and job possibilities also need to be considered.

*Burmese is used for all ethnic groups

## Background

The United Nations High Commissioner for Refugees (UNHCR) estimates that half the 20 million refugees in the world are young people (15-24 years) currently displaced by armed conflict. About one third or approximately 6.6 million are adolescents aged 10-19 [1]. Youth have sexual and reproductive health needs that may differ from adults, but they remain poorly understood and underserved [2]. In situations of conflict, the absence of appropriate services and trained providers is a major barrier to ensuring young people's right to a healthy and productive life [1,3] and may create permanent problems. There has been little research on reproductive health and quality of life of youth living in refugee camps, particularly in the context of Asia. We aimed to assess young refugee's reproductive health information sources, knowledge, attitude, beliefs and norms as well as their quality of life in this long-term setting. This study was conducted in two refugee camps (Mae Ra Ma Luang used as MRML and Mae La Oon used as MLO) in Thailand, home to about 32 000 Burmese refugees.

## Methods

### Study Area

Since 1976, the civil war between ethnic minorities and the military regime in Myanmar (Burma) has resulted in a mass influx of refugees and migrants into neighbouring Thailand, Bangladesh and India. Flight to these countries continues for those forcibly expelled from the conflict zones. The situation can be regarded as a forgotten crisis with a complex political origin [4]. Currently, an estimated 400 000 refugees from Myanmar reside in refugee camps or in villages along the north-western border inside Thailand. Of the 140 000 people living in the nine refugee camps, 43 000 (30%) are young people between 10-24 years [5]. They have grown up isolated in a closed setting with little access to the outside world with a notable systematic aid dependency by receiving shelter, food, health services and education from the Thai government and the international community [6]. Formal education is limited to ten years of schooling, while job opportunities and access to universities is very limited. Traditional social norms and religion (Burmese Buddhist, Christian or Animists) strongly influence the daily life and behaviour of the refugee population.

The two study camps MRML and MLO are located 80 km south of Mae Sariang town, deep in the tropical forest on the Thailand-Myanmar border. Access to the camps is difficult during the monsoon season from June to October. Health and nutrition services are provided mainly by refugee health workers trained by international NGO's.

### Health Aspects

Health services in the two refugee camps are concentrated on communicable disease control. Reproductive health services are covering mainly pre- and post-natal care and family planning for married couples. The needs of unmarried youth for issues related to reproductive and sexual health are not fully addressed, while the refugee society does not allow them access to condoms or reproductive education or other sexual health services, for fear that information and access may promote promiscuity.

### Assessment techniques

A stratified randomized cross-sectional survey was carried out to assess knowledge of reproductive health issues, attitude, beliefs and norms and quality of life of young refugees aged 15 - 24 years. The survey format and questions were based on an adolescents' sexual behaviour questionnaire developed by Cleland et al [7]. As recommended by Cleland et al, the questionnaire was pre-tested, edited and modified to our setting. Qualitative methods were used to extend and triangulate findings from the survey. The tool is flexible with each question basically standing on its own. It has been used widely by experienced teams and is therefore considered to have content or consensual validity i.e. "a number of experts agree that a measure is valid" [8]. The questionnaire was translated from English into Karen and back translated to English by two independent translators and discrepancies were verified. A topic guide was developed for the qualitative interviews to explore; youth daily life and education in the camps; their main reproductive and health concerns; future life expectations and possible solutions. Six of the men and seven women allowed their interviews to be tape-recorded. The definition for Quality of life' in this study: related to assessment of happiness, pleasure, feeling of satisfaction [9], feelings of optimism/hope, and life as meaningful [10].

### Procedures

Based on an expected prevalence of basic knowledge of major reproductive health issues of 50%, and absolute precision set at 5% (d = 0.05), it was estimated that a sample size of 403 youth, rounded to 400, was required; we allowed for a 5% drop out or refusal to participate. The primary sampling frame was a list of 5,183 households and school dormitories.

The stratified two-stage random sampling technique was carried out using SRS (Simple Random Sampling) software. A sample proportional to section size was generated using random numbers, to select 400 households and school dormitories in the first stage. In the second stage all youth aged 15-24 years who lived in the 400 randomly selected houses were listed with the support of the community health workers; in houses and school dormitories with more than one eligible person, one young person from the household list was randomly selected. If a selected house did not provide an eligible person within the age range 15-24, a further household was randomly selected.

For the one-to-one semi-structured interviews, youth were purposely selected by a health worker and the head master of the school, to include different ages, males and females, and married and unmarried youth; all interviews were documented and analysed using the framework approach [11].

Ethical approval was given by the London School of Hygiene and Tropical Medicine and by the Karen Ethical Committee in Thailand. Verbal consent was obtained by two camp representatives, a teacher and mother.

### Data Analysis

Survey data were analysed using SPSS for Windows software (SPSS Inc, Chicago, Illinois, USA, Version 11.0). Continuous normally distributed variables were described by their mean and standard deviation (SD), and if non-normally distributed, the median (range) was reported. Percentage were given for categorical data and presented in contingency tables or pie charts. Comparison of characteristics of the two camp population samples was made using EPI. Info software (Version 6.04d).

For the multivariate analysis we dichotomized the 10 questions to' correct' and' no- or wrong' knowledge to assess the association between knowledge of reproductive health issues and marital status, taking confounding effects of sex, age group, education, previous sex education and work for pay, into account.

## Results

### Survey questionnaire

The survey was conducted in June 2005 and January 2006 over a period of four days; 397/400 (99.25%) youth participated in the self-administered questionnaires, 217 in MLO- and 180 in MRML camp. As there were no significant differences in socio-demographic characteristics between the two populations, data were pooled for further analysis (Table 1).

**Table 1:** Socio-Economic and Demographic Characteristics by sex; all respondents

Characteristics	Male (%)	Female (%)	Total (%)
	(n = 215)	(n = 182)	(n = 397)
Marital status			
-single	183 (85.1)	127 (69.8)	310 (78.1)
-married	32 (14.9)	55 (30.2)	87 (21.9)
			
Mean Age	18.6 (SD. 18.6)	18.4 (SD 18.4)	18.5 (SD 2.7)
Age Group			
- 15-19	144 (67.3)	122 (67)	266 (67.2)
- 20-24	70 (32.7)	60 (33)	130 (32.8)
			
Education			
- currently in primary school	39 (18.8)	26 (15.1)	65 (17.1)
- currently in secondary school	99 (47.6)	74 (43)	173 (45.5)
- finished/stopped primary school	23 (11)	24 (13.6)	47 (12.4)
- finished/stopped secondary school	47 (22.6)	48 (27.9)	95 (25)
			
Living Condition			
- stay alone/Dormitory	40 (18.6)	22 (12.1)	62 (15.6)
- with parents	120 (55.8)	104 (57.1)	224 (56.4)
- with relatives	30 (14)	13 (7.1)	43 (10.9)
- with spouse	25 (11.6)	43 (23.6)	68 (17.1)
			
Average School fee paid/year	2.6 $US	2.3 $US	2.5$US
			
Religion			
- no religion	2 (0.9)	0	2 (0.5)
- Buddhist	33 (15.3)	29 (15.9)	62 (15.6)
- Baptist	135 (62.8)	124 (68.1)	259 (65.2)
- Roman Catholic	20 (9.3)	18 (9.9)	38 (9.6)
- Islam	0	2 (1.1)	2 (0.5)
- Seventh Day	18 (8.4)	7 (3.8)	25 (6.3)
			
Importance of Religion			
-very important	175 (82.2)	154 (85.1)	329 (83.5)
- important	33 (15.5)	23 (12.7)	56 (14.2)
- not important	5 (2.3)	4 (2.2)	9 (2.3)

Two-thirds of the youth had lived in the camp for more than seven years; one in six for longer than ten years. Alcohol consumption was reported by 18% and the use of illicit drugs by 7.6% (30/397) of the respondents; all were male. No details were provided concerning which kind of drugs the respondents consumed.

### Reproductive Health Information Sources

Ten questions were asked on knowledge and use of family planning method. Questions on sources of information related to reproductive health were addressed to all interviewees (Table 2). Over 60% of both male and female youth reported that they would like to receive information from health workers, but only a third received any. Thirty two percent of young women (43/136) received reproductive health information from their mothers while the more usual source of information for young men was a friend (29/143, 20.3%). Most of interviewees (70.6%, 279/395) asked for more classes on topics including sex education, puberty and relationships.

**Table 2:** Knowledge of pregnancies and contraception; by sex; all respondents

	Male	Female	Total
Is pregnancy possible after	(n = 214)	(n = 182)	(n = 396)
first sexual intercourse?			
- yes	37 (17.2)	39 (21.4)	76 (19.2)
- no	36 (16.8)	23 (12.6)	59 (14.9)
- DK*	141(65.9)	120 (65.9)	261 (65.9)
			
Is pregnancy likely if a woman has sexual intercourse half way between periods	(n = 212)	(n = 181)	(n = 393)
- yes	51 (24.1)	41 (22.7)	92 (23.4)
- no	22 (10.4)	15 (8.3)	37 (9.4)
- DK	139 (65.6)	125 (69)	264 (67.2)
			
Women can take pill daily	(n = 215)	(n = 182)	(n = 397)
-yes	33 (15.3)	51 (28)	84 (21.2)
-No	9 (4.2)	7 (3.8)	16 (4)
-DK	173 (80.5)	124 (68.1)	297 (74.8)
			
Condom can be used during sex	(n = 215)	(n = 182)	(n = 397)
-Yes	79 (36.7)	71 (39)	150 (37.8)
-No	19 (8.8)	3 (1.6)	22 (5.5)
-DK	117 (54.4)	108 (59.3)	225 (56.7
			
You know where to get pill?	(n = 214)	(n = 182)	(n = 396)
-Yes	64 (29.9)	64 (35.2)	128 (32.3)
-No	150 (70.1)	118 (64.8)	268 (67.7)
			
You know where to get condoms?	(n = 214)	(n = 181)	(n = 395)
-Yes	89 (41.6)	50 (27.6)	139 (35.2)
-No	125 (58.4)	131 (72.4)	256 (64.8)
			
You know where to get injection?	(n = 214)	(n = 162)	(n = 395)
-Yes	61 (28.5)	59 (36.4)	120 (30.4)
-No	153 (71.5)	122 (75.3)	275 (69.6)

These results indicate that only 19% (95%CI 15.6%-23.4%) of the youth were aware that first sex could result in pregnancy. About 23% (95%CI 19.5%-27.8%) knew that sex half way between periods could lead to pregnancy. The role of condoms was known by only 37.8% (95%CI 31.1%-42.7%) and where to obtain them by only 35.2% (95%CI 30.6%-40%). Multivariate analysis identified that marital status was strongly associated with sexual health knowledge in young refugees, with the odds of having relevant knowledge of reproductive health being up to six times as high for married young people as for those who were unmarried, after adjusting for socio-economic and demographic factors

### Attitude towards Condoms

The questions to elicit knowledge of and attitude towards condoms consisted of 12 statements using a three-point Likert scale [12]. Responses were obtained from 394 (99.5%) respondents. All groups tended to consider it important to use condoms in a protective role against HIV/AIDS and sexually transmitted infections or prevention of pregnancy (205/393; 52.2%), with married youth more likely to consider this than the unmarried. Nearly half the young refugees favoured the use of condoms for casual relationships (184/393; 46.7%). When it came to practical aspects of obtaining condoms from the clinics, or using condoms, four in five married men (26/32; 81.3%) said they would feel embarrassed, as did two in three married women (38/55; 69.1%); the single men (107/180; 59.4%) and women (45/126; 35.7%) were less embarrassed.

### Beliefs and Norms towards sexuality

The following section on beliefs and norms towards sexuality was addressed to all interviewees and consisted of 12 questions.

More men than women considered premarital sex to be acceptable. However just over half the interviewees thought that both boys and girls should remain virgins until marriage. It was of concern that one in three of both, men and women, thought it acceptable for a young man to force a woman to have sex if he loved her, and 18% men and 15% women considered it acceptable for a boy to hit his girlfriend (Table 3).

**Table 3:** Beliefs and Norms towards sexuality; by sex; all respondents

	Male	Female	Total
Do you believe it's alright for unmarried boys and girls to meet	(n = 214)	(n = 182)	(n = 396)
- yes	150 (70.1)	96 (52.7)	246 (62.1)
- no	15 (7)	14 (7.7)	29 (7.3)
- DK	49 (22.9)	72 (39.6)	121 (30.6)
			
Do you believe it's alright for unmarried boys and girls to kiss, hug and touch	(n = 210)	(n = 182)	(n = 392)
-Yes	122 (58.1)	78 (42.9)	200 (51)
-No	37 (17.6)	52 (28.6)	89 (22.7)
-DK	51 (24.3)	52 (28.6)	103 (26.3)
			
Do you think it is OK if unmarried youth have sex if they love each other	(n = 213)	(n = 182)	(n = 395)
-Yes	97 (45.5)	52 (28.6)	149 (37.7)
-No	47 (22.1)	63 (34.6)	110 (27.8)
-DK	69 (32.4)	67 (36.8)	136 (34.4)
			
Is it OK if sometimes a boy force a girl to have sex if he loves her	(n = 214)	(n = 182)	(n = 396)
-Yes	65 (30.4)	60 (33)	125 (31.6)
-No	40 (18.7)	41 (22.5)	81 (20.4)
-DK	109 (50.9)	81 (44.5)	190 (48)
	(n = 214)	(n = 182)	(n = 396)
			
Do you think most girls who have had sex before marriage regret it?			
-yes	91 (42.5)	93 (51.1)	184 (46.5)
-no	11 (5.1)	4 (2.2)	15 (3.8)
-DK	112 (52.3)	85 (46.7)	197 (49.7)
			
Do you think most boys who have had sex before marriage regrets it?	(n = 211)	(n = 180)	(n = 391)
-yes	92 (43.6)	73 (40.6)	165 (42.2)
-no	18 (8.5)	14 (7.8)	32 (8.2)
-DK	101 (47.9)	93 (51.6)	194 (49.6)
			
Do you believe girls should remain virgin until marriage?	(n = 214)	(n = 181)	(n = 395)
-yes	103 (48.1)	103 (56.9)	206 (52.1)
-no	23 (10.7)	7 (3.9)	30 (7.6)
-DK	88 (41.1)	71 (39.2)	159 (40.3)
			
Do you believe boys should remain virgin until marriage	(n = 208)	(n = 180)	(n = 388)
-yes	109 (52.4)	103 (57.2)	212 (54.6)
-no	22 (10.6)	8 (4.4)	30 (7.7)
-DK	77 (37)	69 (38.3)	146 (37.6)
			
Is it justifiable for a boy to hit his girlfriend	(n = 208)	(n = 182)	(n = 390)
-yes	37 (17.8)	27 (14.8)	64 (16.4)
-no	61 (29.3)	58 (31.9)	119 (30.5)
-DK	110 (52.9)	97 (53.3)	207 (53.1)
			
Men need sex more frequently than women	(n = 211)	(n = 181)	(n = 392)
-yes	70 (33.2)	55 (30.3)	125 (31.9)
-no	12 (5.7)	9 (5)	21 (5.3)
-DK	129 (61.1)	117 (64.7)	246 (62.8)
			
Do you think that one night stands are OK	(n = 213)	(n = 182)	(n = 395)
-yes	12 (5.6)	4 (2.2)	16 (4.1)
-no	117 (54.9)	101 (55.5)	218 (55.2)
-DK	84 (39.4)	77 (42.3)	161 (40.7)

### Marriage Practices

The following section relates to married interviewees only and consists of eight questions. Questions related to age of marriage, first sexual intercourse and how the marriage was formed. Almost all the young married refugees (84/87) responded to the questions. The average age of marriage for men was 20 years (SD 2.2) and for women 18 years (SD 2.2).

Of the female youth, 61.5% (32/52) were married by the age of 18. The average age of first sexual intercourse among married men was 19.7 (SD. 2.1) years and among married women 17.9 years (SD. 2.3). More than half (54.2%) had their first sexual experience before marriage (Table 4). The reason to marry was given by 57.8% (48/83) as having been found to have had sex. Twenty five per cent (21/84) reported that young people were often forced to marry; 52.4% (44/84) reported that it sometimes happened. When asked whose decision it was to marry, 82.1% (69/84) reported that it was their own decision to marry. Two third of those married, felt they did not marry at the right age (65.1%, 54/83).

**Table 4:** Age at marriage, first sex and premarital sex; married respondents by sex

	Male (%)	Female (%)	Total
Age at marriage	(n = 32)	(n = 52)	(n = 84)
14 yrs	0	2 (3.8)	2 (2.4)
15-19 yrs.	15 (46.9)	37 (71.2)	52 (61.9)
20-24 yrs.	17 (53.1)	13 (25)	30 (35.7)
			
Age at first sex	(n = 32)	(n = 51	(n = 83)
14 yrs.	0	4 (7.8)	4 (4.8)
15-19 yrs.	19 (59.4)	36 (70.6)	55 (66.3)
20-24 yrs.	13 (40.6)	11 (21.6)	24 (28.9)
			
Were you married before first sex	(n = 32)	(n = 51)	(n = 83)
- yes	11 (34.4)	27 (52.9)	38 (45.8)
- no	21 (65.6)	24 (47.1)	45 (54.2)

More than half said they (or their partner) did not use any contraception at the time of the interviews (46/84; 54.8%). When we asked all participants what young people require in terms of health services 65.9% (259/393) asked for more health education followed by special services for young women (92/393, 23.4%).

### Quality of Life and expectation for their future life

**Figure 1:**
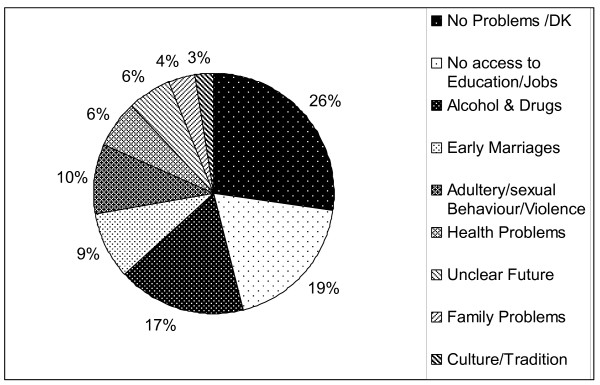
**Main Problems perceived and ranked by the varies themes; all respondents**.

Six questions related to young people's perception of their quality of life and their expectations for their future life. Figure 1 summarizes answers from an open ended question on the main problems they perceived in their lives. This question was answered by 99.5% (395/397) of the youth.

The limited access to further education, and concern about alcohol and drug abuse, were regarded as the main problems (Figure 1) but what they would see as the solutions to these problems remained unclear or without a solution (153/396, 38.6%). One third (124/396, 31.3%) suggested their life would be improved by more access to education and jobs and that refugee authorities should provide these solutions (106/396, 26.8%). A few suggested that external support through donors and NGO's could ameliorate the major problems.

Most youth indicated that what they liked most in their life was reading books or going to school (51.8%, 205/396), while unity among the Karen and other ethnic groups was also considered important (37.4%, 148/396). Quarrels in the families and gossip were most disliked (46.8%, 185/395), while being a refugee and having no freedom was reported as a bigger problem for single (31.8%, 98/308) interviewees than for married (17.2%, 15/87). Health problems were the major problem of 24.6% (97/395) of the youth. Almost half (45.3%, 107/236) mentioned headache and dizziness followed by malaria (33.9; 80/236) as the major reason seeking health services in the last 12 months. When asked if their life was meaningful to them, the vast majority (97.7%, 383/392) said yes. Almost half wanted more education and job opportunities within the next five years (186/393, 47.3%), and about a third wanted better health (136/393, 34.6%). One in five youth wanted freedom for their country and to be able to move freely in the future (71/393, 17.1%).

### Results of the semi-structured interviews

The qualitative interviews were carried out in January 2006. Seven of the participants were male and twelve were female. One of the boys and four of the girls were married. Ten of the participants were in the age group 15-19, and nine were in the age group 20-24. The average length of stay in the camp was seven years, ranging from one to 14 years. Seven of the 14 singles lived with their parents; the remaining seven lived in one of the six dormitories. The five married youths had left school as soon as they married. All regretted not being able to finish their education. They married between 14 to 20 years. All felt they did not marry at the right age. Three said they married because they had pre-marital sexual relationships and two were forced to marry due to financial constraints and pressure from their families.

### Knowledge of reproductive health issues

We asked the participants if they had been taught about the period of adolescence, when the body of a girl and a boy is changing. Both, women and men reported they had been taught some basics; with the women getting their information from their mothers and the men from a friend or in one of the advanced schools. A few had been taught nothing about it.

Women tend to be informed by their mothers about body changes, but apparently little information has been given on menstruation issues or sexual relationships; also the young women were not aware that first sex could result in pregnancy.

We asked the married participants if they knew before they married or had their first sex, that first sexual relationships could lead to pregnancy; a young man said he did know but the women did not. None of the unmarried participants knew that first sex could lead to pregnancy. Two men said they knew that condoms could prevent pregnancy and one 20 year old married participant reported that he had been worried the first time he had sex with his girl friend that she would become pregnant. He was aware that first sex could result in pregnancy, but had never seen a condom nor knew how to access or use it.

*Quote by a young woman (22 years): I got pregnant when I was 14 years because I did not know when I had my first sexual relationship that it could result in pregnancy; that is why I got pregnant"*.

*Quote by a young woman (17 years): I did not know that first sex can result in pregnancy but my husband knew; but he said he really loves me and that's why we did not use any contraception. I did not want to have children at that time but I got pregnant*.

### Health Issues

Most female interviewees complained about menstruation problems and three women reported that they often wore wet underwear especially during that time. They reported that they did not have enough underwear for changes, and that they had to dry their garments inside the latrines to avoid walkways where the men were likely to pass.

*Quote by a young woman (16 years): "I am lucky not having my monthly menstruation regularly otherwise I would be in trouble because I have not enough garments to change"*.

### Quality of Life

We asked all interviewees a broad question about how meaningful their life was to them. Most reported that their life was not meaningful, of whom a young woman and man said they felt hopeless or not happy. The major reason was because they had no work and therefore could not support their families. They expressed it by saying:

*"We cannot contribute anything to the community"*.

Most worried about their unclear future, having no money and depending on the international community and the Thai government, that they did not live in their home country, and had limited opportunities for further studies or job opportunities.

*"Living in the dormitory and having no freedom in my life yet and most likely no job in the future, my life is meaningless"*.

*"My life would be meaningful if I get work and a free life in Myanmar"*.

*"I cannot stand on my own feet and cannot support my family"*.

*"I live in the dormitory and I feel good having an opportunity to study in the camp schools; other people in my village in Myanmar have no chance to receive education; this makes me sad and depressed". Sometimes I get headaches from this and sometimes sought help from friends or from the NGO counsellor in the camp". I believe that there are more boys than girls having similar problems to my.....Men have to think more about the future than women do"; "men will lead the family in the future", also many boys are unhappy because they have a girl friend and they do not know how to meet her"*.

Quote by a young woman (22 years):

*"I would like more education and a job in the future; I married when I was 14 and had to stop my education; there is also little information available for refugees who want to resettle in a third country while the UN should provide more information on the resettlement countries"*.

*"In the future I want to live in a peaceful place where there is no fighting and where I and my family can stay without being afraid of being killed"*.

In summary, the life of the young refugees in the camps is restricted and limited in terms of movement; premarital sex and financial constraints may lead to premature marriage for young people which hinders further education, since all had to drop out from school. Most interviewees did not have the basic knowledge that first sex could lead to a pregnancy. Almost all the young people reported that their life lacked meaning; most felt bored and unhappy, with no work, no income and not able to contribute to the community. Adding to that was their unclear future in the camps, in Myanmar or in a third country.

## Discussion

This research addressed issues related to reproductive health and quality of life and aimed to identify gaps and needs of the refugee youth affected by conflict and living in this long-term settlement camps in Thailand. These issues have not been seriously considered until now.

Access to reproductive health information, education and services was very limited in the two camps evaluated, and youth's knowledge of sexual and reproductive health and contraception was extremely low. Similar to a study in Afghanistan [13] the consequences of unprotected sexual intercourse were not well understood by a substantial proportion of youth in the camp and confirmed in the one to one interviews. It is often assumed that respondents answer self-administered questionnaires, such as used in this study, more truthfully, although there is no conclusive evidence on this. However, a large proportion of mainly unmarried youth responded with 'don't know', which can be considered as having insufficient knowledge. Nevertheless, it is not clear if the refugee youth felt uncomfortable or confused to answer questions on pregnancy or contraception in this study. No study of a similar population has been found to further interpret these high 'don't know' responses.

It has become clear that sexual health education in this long-term settlement is a particular problem, which needs to be addressed; young refugee's misconceptions on important questions relating to reproductive health issues have caused them to pay a high price when they get sexually engaged, as they are then *forced *to marry and lose the one opportunity for education. Bott and Jejeebhoy [2] and Jejeebhoy et al [14] reported that in Asia, parents themselves lack knowledge, feel embarrassed and prefer to leave issues of reproductive health to textbooks and teachers. The limitation for reproductive health information through schools as well as parental embarrassment explain why the large majority of interviewees say they would prefer to receive reproductive health education from health workers rather than from teachers or family members. Health workers working with the NGO's may be perceived as neutral as well as knowledgeable, and young people probably expect more tolerance and openness on a subject that has been taboo for them. In a global study on reproductive health issues [15], health workers were regarded as credible sources of information by young people and their parents. Studies in England have also shown health workers to be the source of preference among adolescents for promoting a healthy life style [16,17]. Youth in this study desired more information and services, as proposed by the Cairo declaration [18].

According to camp official health data, family planning is used by 12% on average, which is very low compared to non-camp situations. This might be related to an overall cultural high value on having many children especially where there is a strong philosophy of replacing those who have been lost in wars. We do not know if that is the case for the population under study. Evidence from other refugee camps or internal displaced settings (IDP) indicates that young people become sexually active at an earlier age than do those living under normal non-camp conditions [19,20]. This behaviour might be a mechanism linked with prospects of a hopeless and desperate future. Globally, pregnancy and childbirth in adolescent girls are associated with high rates of mortality and morbidity [21,22].

It is common for unmarried pregnant young women to not attend antenatal or other health care services due to embarrassment for the young women and their families. This reflects similar concerns in refugee camps in Tanzania [23]. Youth and single adults are not supposed to have premarital sex or to need reproductive health services. However, the age-specific pregnancy rate (per 1000) among those aged 15-19 was 60 per 1000 (41/687) in MLO camp and 80 per 1000 (45/562) in MRML camp in 2006 (camp data). We were not able to compare the camps age-specific pregnancy rate of 60-80 per 1000 in youth aged 15-19 with other similar settings; but to put the rate into some perspective, according to Singh and Darroch [24] this number of pregnancies per youth population aged 15-19 is considered medium to high compared to pregnancy rates among youth in Europe and the USA. India reported a pregnancy rate of 39 per 1000 (2006) and in Cambodia of 30 per 1000 youth (2005) in the same age group [25].

There would appear to be changing attitudes towards relationships among the youth, away from the traditional expectation of Karen and Burmese society. Religion and traditions remain important and strong and are the basis of the strict adult sexual code; this tie is apparently loosening for the youth. Traditions and religion may however be considered to be partly protective and used as a coping mechanism in this society [26] by providing rules and norms steering young people away from pre-marital sex. According to Belak [27] it can be said that religion has a strong influence on cultural and traditional norms and behaviour in both Burmese and Karen society, which are intertwined. Belak pointed out that "Burmese Buddhism" has influenced Christian and Animist traditional norms as practiced in Myanmar. The cultural norms are to a large extent common to the different religious groups in Myanmar.

Considering the quality of life, there were differences between the responses in the quantitative and qualitative research, related to the question of how meaningful their life is. In the qualitative interviews most said they suffered greatly from boredom and unhappiness as they had no possibility of contributing to society. In the quantitative survey the participants responded more positively, with most saying that their life was meaningful. This question needed in depth probing and the qualitative interviews are likely to be more informative.

It is possible that their life is better in some ways than that of youth living in other refugee camps or countries in South East Asia. Indeed, the education opportunities in these camps are better than for example for the general non-camp Nepalese youth where 26% of the boys and 51% of the girls aged 15-19 are illiterate[2]. But when these Burmese refugee youth finish school, their grade is not acknowledged in Thailand, nor in Myanmar or elsewhere. Moreover, they live in a totally confined setting where work and livelihood opportunities are almost non-existent, where refugees depend fully on international aid and where youth feel that they do not and cannot contribute to society.

The findings are likely to be generalizeable to other refugee camps along the border area since all these camp populations, are similarly ethnically diverse (Karenni, Burmese or Mon), coming from Myanmar with very similar cultural and traditional backgrounds.

The implications for policy change are clear. The current developments where some refugees are offered resettlement in a third country provide additional strong arguments to be considered by the refugee leadership, the United Nations High Commissioner for Refugees, the donors and the aid agencies. The youth being resettled will be even more exposed to issues related to sexuality. To provide young refugees with necessary and effective information and services for their future and to equip them with skills for their transition into adulthood should be a mandatory policy set by the stakeholders.

## Competing interests

The authors declare that they have no competing interests.

## Authors' contributions

MTB has been the principal investigator, designed and lead the study, analysed the data, interpreted the findings and wrote up the article. JT and ES supported the design of the study, interpreted the data and supported the writing of the article and edited the final text. KH translated the questionnaire and entered the data. WK led the field work; WK, KH and NN carried out the semi structured interviews. SH and VIC supported the analysis and interpretation of the data as well edited the final text. All authors read and approved the final manuscript.

## Authors' informations

1. Independent Researcher

MTB - Corresponding Author; works currently with the European Commission Humanitarian Office (ECHO) in Bangkok, Thailand. The study was part of her doctoral degree in Public Health at the London School of Hygiene and Tropical Medicine, London, UK

mtbenner@gmx.de

WK - Reproductive Health Coordinator for Malteser International;

rchc.msr1@malteser-international.org

KH - former Laboratory Supervisor for Malteser International; left for resettlement

kyi_htwe@yahoo.com

NN - former Health Promotion Coordinator for Malteser International;

flowernantaree@yahoo.com

2. London School of Hygiene and Tropical Medicine, London, UK

JT - Emeritus Professor at the London School of Hygiene and Tropical Medicine; joy.townsend@lshtm.ac.uk

ES - Senior Lecture in Public Health and Humanitarian Aid;

egbert.sondorp@lshtm.ac.uk

SH -PhD Student; saowalak.hunnangkul@lshtm.ac.uk

3. Faculty of Medicine Siriraj Hospital, Mahidol University, 10700 Bangkok, Thailand

SH - Biostatistician

4. SHOKLO Malaria Research Unit, PO Box 46, Mae Sot, Tak, Thailand

VIC - Epidemiologist; verena@shoklo-unit.com
